# Unobtrusive Nocturnal Heartbeat Monitoring by a Ballistocardiographic Sensor in Patients with Sleep Disordered Breathing

**DOI:** 10.1038/s41598-017-13138-0

**Published:** 2017-10-13

**Authors:** Matthias Daniel Zink, Christoph Brüser, Björn-Ole Stüben, Andreas Napp, Robert Stöhr, Steffen Leonhardt, Nikolaus Marx, Karl Mischke, Jörg B. Schulz, Johannes Schiefer

**Affiliations:** 1 Department of Cardiology, Pneumology, Angiology and Intensive Care Medicine, University Hospital RWTH Aachen, Pauwelsstr. 30, 52074 Aachen, Germany; 2 Philips Chair for Medical Information Technology, Helmholtz-Institute, RWTH Aachen, Pauwelsstr. 20, 52074 Aachen, Germany; 3 Department of Neurology, University Hospital RWTH Aachen, Pauwelsstr. 30, 52074 Aachen, Germany; 4 Jülich Aachen Research Alliance (JARA) – JARA-Institute Molecular Neuroscience and Neuroimaging, FZ Jülich and RWTH University, Aachen, Germany

## Abstract

Sleep disordered breathing (SDB) is known for fluctuating heart rates and an increased risk of developing arrhythmias. The current reference for heartbeat analysis is an electrocardiogram (ECG). As an unobtrusive alternative, we tested a sensor foil for mechanical vibrations to perform a ballistocardiography (BCG) and applied a novel algorithm for beat-to-beat cycle length detection. The aim of this study was to assess the correlation between beat-to-beat cycle length detection by the BCG algorithm and simultaneously recorded ECG. In 21 patients suspected for SDB undergoing polysomnography, we compared ECG to simultaneously recorded BCG data analysed by our algorithm. We analysed 362.040 heartbeats during a total of 93 hours of recording. The baseline beat-to-beat cycle length correlation between BCG and ECG was *r*_*s*_ = 0.77 (n = 362040) with a mean absolute difference of 15 ± 162 ms (mean cycle length: ECG 923 ± 220 ms; BCG 908 ± 203 ms). After filtering artefacts and improving signal quality by our algorithm, the correlation increased to *r*_*s*_ = 0.95 (n = 235367) with a mean absolute difference in cycle length of 4 ± 72 ms (ECG 920 ± 196 ms; BCG 916 ± 194 ms). We conclude that our algorithm, coupled with a BCG sensor foil provides good correlation of beat-to-beat cycle length detection with simultaneously recorded ECG.

## Introduction

Sleep disordered breathing (SDB) is widespread^1,2^ and characterized by pathological breathing episodes possibly leading up to complete apnea. Through fragmented sleep architecture, untreated SDB can result in reduced quality of sleep and increased morbidity^3^. SDB is associated with an increased risk for obesity^4^, abnormal body and limb movements, fluctuating heart rates and risk for the development of cardiac arrhythmias^5^. Thus, an unobtrusive device for long-term heartbeat measurement is desirable for this group of sleep disordered individuals and other patients who are at risk for arrhythmia^6^.

The gold standard for heartbeat diagnosis is the electrocardiogram (ECG), but novel unobtrusive systems are the focus of research looking for alternatives for heart rate monitoring^7^. These include wearable electrodes^8^, smartphone applications^9^, photoplethysmographic-^10^, unobtrusive-^11^ or mechanical sensors^12,13^, developed as an alternative for heart rate measurement to address future aspects of individualized diagnosis and therapy. While ECG measures cardiac electrical activity, a ballistocardiography (BCG) foil registers the mechanical vibrations caused by cardiac activity. This method came into focus at the end of the 19^th^ century^14^. Initially, the signal of mechanical vibrations offered reliable information about respiration rate^15–17^ and cardiac contractility^18^. Thanks to advances in sensor and algorithm technology, analysis of the cardio-mechanical signal is now able to deliver additional information regarding heart rate^19–21^ or pulse transit time^22^ for cuff-less blood pressure monitoring.

**Figure 1:**
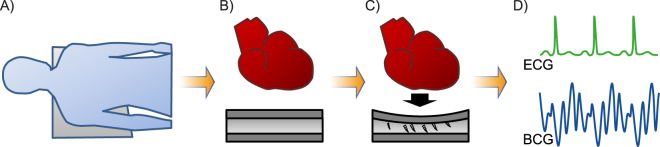
BCG measurement and correlation with a synchronized recorded ECG. (**A**) BCG foil is positioned under the patient**’**s chest. (**B**,**C**) Mechanical cardiac activity induces a charge shift in the BCG foil. (**D**) A BCG signal is recorded and compared to a simultaneously recorded ECG.

A BCG foil can be positioned invisibly beneath the bed sheets (Fig. 1A). The mechanical vibration of each heartbeat is recorded and interpreted by our novel algorithm (Fig. 1B,C) as a surrogate for the heartbeat cycle length and compared to a simultaneously recorded ECG during an in-laboratory polysomnography (PSG) (Fig. 1D).

In preceding investigations, we demonstrated that short term beat-to-beat analysis for known episodes of atrial fibrillation and sinus rhythm by a BCG sensor are feasible^23^. This study now extends our previous approach by applying autonomous beat-to-beat recording with robust and reliable analysis for hours, live feed of the acquired BCG signal similar to a telemetric monitoring system, automated exclusion of episodes with no signal or low signal quality and an increase in the accuracy of cycle length estimation by a computed quality index (QI). In addition, this long-term approach should help to identify potential fields of application, for which kind of heartbeat surveillance the sensor may be suited best. We selected a SDB cohort to test the performance of our novel algorithm as it represents a difficult measurement situation caused by patient related physical constraints including obesity, restless sleep as well as a highly fluctuating heart rate.

Thus, the main objective of this study was to test the feasibility of long-term beat-to-beat cycle length calculation by means of BCG.

## Results

A total of 93 hours with 362.040 identified heartbeats in 21 BCG recordings were analysed. Mean age of the included patients was 50 ± 13 years and 19 of the participants were male (91%) (Table 1). The patient cohort was generally overweight with a mean Body-Mass-Index (BMI) of 33.8 ± 9.6 kg/m^2^. All patients suffered from snoring and symptoms related to sleep disorder breathing. Preliminary assessment included a mean Epworth sleepiness scale (ESS) score of 13 ± 5 and an Apnea-Hypopnea-Index (AHI) of 40.9 ± 21.7 before PSG.

**Table 1:** Baseline data of the study group.

		Mean ± Standard Deviation (SD)	N (%)
**Study group**			
Age		50 ± 13	
Sex	Male		19 (91%)
Smoker	Yes		4 (19%)
Height [cm]		1.77 ± 0.09	
Weight [kg]		107 ± 33	
BMI [kg/m^2^]		33.8 ± 9.6	
AHI [/h]		40.9 ± 21.7	
ESS		13 ± 5	
**Polysomnography**			
RDI [/h]		26.9 ± 23.8	
Mean Oxygen Saturation [%]		91 ± 4.2	
Minimal Oxygen saturation [%]		77.1 ± 10.9	
No sleep apnea syndrome			5 (24%)
Mild sleep apnea syndrome			3 (14%)
Moderate sleep apnea syndrome			3 (14%)
Severe sleep apnea syndrome			10 (48%)

During PSG, the average oxygen saturation was 91 ± 4.2%, while minimal saturations of 77.1 ± 10.9% were recorded. Sleep apnea syndrome was diagnosed in 16 (76%) patients (Table 1).

**Figure 2:**
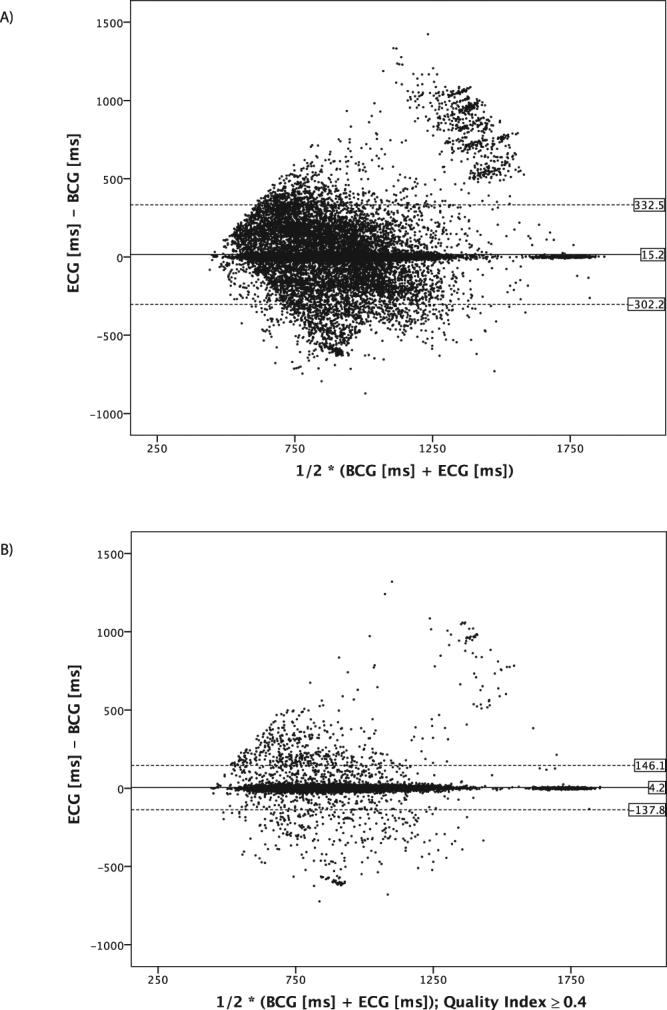
Bland-Altman plots of the analysed heartbeats. To generate the plots, 10% of all recorded heartbeats (n = 326040) were randomly selected to avoid clutter. (**A**) Mean differences of all analysed heartbeats for BCG and ECG. The mean difference of simultaneously recorded heartbeats for ECG and BCG is 15 ± 162 ms. (**B**) Mean differences of all analysed heartbeats for BCG and ECG filtered with QI ≥ 0.4. The mean difference for simultaneous recorded heartbeats is 4 ± 72 ms after filtering. By filtering for better signal quality, the BCG cycle length detection improves accuracy at the cost of the absolute number of analysable heartbeats.

The baseline correlation of the estimated BCG cycle length with the simultaneously recorded ECG was *r*_*s*_ = 0.77 (n = 362040) with a mean absolute difference between ECG and estimated heartbeat cycle length by the BCG algorithm of 15 ± 162 ms (heartbeat cycle length: ECG 923 ± 220 ms vs. BCG 908 ± 203 ms) (Fig. 2A). By calculating the signal quality of each identified heartbeat in the BCG signal, the QI offered the possibility to filter heartbeats of poor quality at the cost of reducing the absolute number of analysable heartbeats (Table 2). Filtering heartbeats of poor quality resulted in an improved correlation coefficient and a more precise cycle length measurement. By raising the QI to ≥ 0.4, the correlation coefficient improved to *r*_*s*_ = 0.95 (n = 235367) with a mean difference in heartbeat cycle length of 4 ± 72 ms (ECG 920 ± 196 ms vs. BCG 916 ± 194 ms) (Fig. 2B).

**Table 2:** Analysed RR intervals by the BCG algorithm and correlation coefficient of the simultaneously recorded ECG filtered by QI and corresponding mean ECG, BCG cycle length and the absolute difference between ECG and BCG cycle length.

Quality Index [au]	n =	Percentage [%]	*r*_*s*_ =	Mean ± SD ECG [ms]	Mean ± SD BCG [ms]	Mean ± SD (ECG – BCG) [ms]
≥0.1	362040	100	0.77	924 ± 220	908 ± 203	15 ± 162
≥0.15	361897	99.9	0.77	924 ± 220	908 ± 203	15 ± 162
≥0.2	358126	98.9	0.8	924 ± 220	908 ± 203	15 ± 161
≥0.25	338193	93.4	0.81	924 ± 217	909 ± 201	15 ± 151
≥0.3	302529	83.6	0.85	923 ± 210	911 ± 198	12 ± 129
≥0.35	266041	73.5	0.91	922 ± 202	913 ± 195	8 ± 101
≥0.4	235367	65	0.95	920 ± 196	916 ± 194	4 ± 72

**Figure 3:**
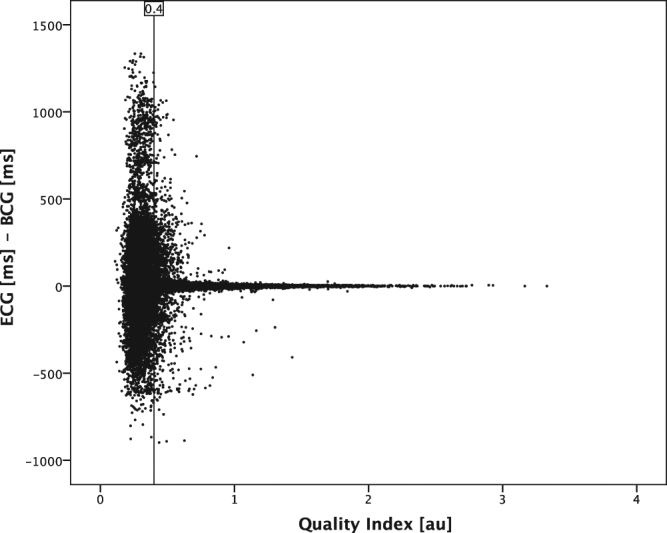
Scatter plot of QI and corresponding differences between analysed cycle length in ECG and estimated cycle length by BCG algorithm. To avoid clutter, randomly selected 10% of all analysed heartbeats are displayed. The scatter plot demonstrates: The higher the QI, the lower the difference between ECG and BCG measurement. An arbitrary cut-off line at the QI value of 0.4 is shown, indicating a correlation coefficient *r*_*s*_ = 0.95 for the data points to the right of this cut-off line.

We next divided all analysed heartbeat lengths into quartiles according to the heart rate of the measured ECG cycle length (Table 3). The QI was highest for the two middle quartiles with a QI of 0.63 ± 0.35 and 0.67 ± 0.35, respectively. Accordingly, the mean difference between the BCG and ECG cycle length in the beat-to-beat analysis was the lowest for the mid-quartiles 11 ± 100 ms and 11 ± 94 ms, respectively. By filtering for heartbeats with better signal quality, the quartiles with slow (1005–2000 ms) and fast (330–795 ms) cycle length were filtered at a higher rate as compared to the quartiles in the middle with an improvement in the estimation of cycle length and QI (Table 3). Distribution of the recorded heartbeats in the context of the calculated QI is visualized in Fig. 3. By adding an arbitrary line plotted at a QI of 0.4, the correlation coefficient of heartbeats right of the line was *r*_*s*_ = 0.95 (n = 235367). The higher the QI, the smaller the absolute difference between the recorded ECG cycle length and the corresponding estimated BCG cycle length, leading to clutter around the x-axis for heartbeats with a high QI.

**Table 3:** All analysed heartbeats were classified by cycle length in 4 similar sized groups. Second and third quartile group show best accordance in mean absolute difference between BCG and ECG and the resulting QI. The group with low or high heart rate show a poorer agreement. By filtering heartbeats with lower signal quality, the accordance could be improved especially in the quartiles with low and high cycle length compared to the two middle quartiles with normal cycle length.

Quartiles by cycle length				
Cycle length [ms]	1005–2000	895–1000	800–890	330–795
Heartbeats [n]	92204	91859	89480	88497
ECG [ms]	1191 ± 238	945 ± 31	846 ± 27	699 ± 75
BCG [ms]	1115 ± 215	933 ± 105	835 ± 97	739 ± 140
Difference ECG – BCG [ms]	76 ± 236	11 ± 100	11 ± 94	−40 ± 148
Quality Index [au]	0.56 ± 0.32	0.63 ± 0.34	0.67 ± 0.35	0.57 ± 0.35
Quality Index≥0.4				
Heartbeats [n]	54730	63728	66041	50868
Percentage [%]	59	69	74	58
ECG [ms]	1172 ± 220	945 ± 31	846 ± 27	714 ± 69
BCG [ms]	1155 ± 218	940 ± 55	842 ± 50	723 ± 91
Difference ECG – BCG [ms]	16 ± 106	4 ± 45	4 ± 43	−9 ± 82
Quality Index [au]	0.74 ± 0.3	0.78 ± 0.32	0.8 ± 0.32	0.78 ± 0.34

**Figure 4:**
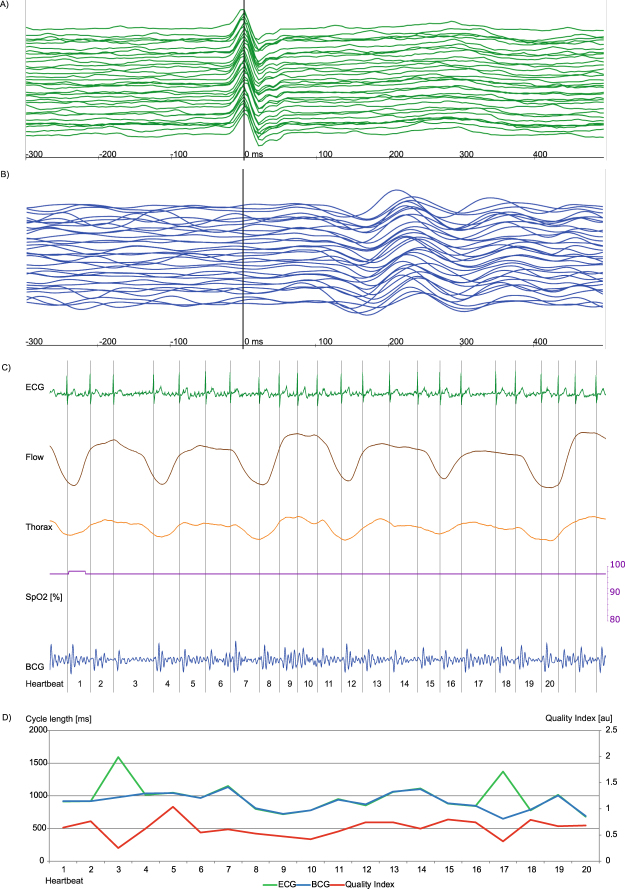
Episode of AF with good signal correlation, for heartbeat 3 and 17 the BCG algorithm estimated a wrong cycle length due to a hampered signal indicated by a low QI (red line, Fig. 4d). Each signal is visualized by a unique colour: ECG (green), BCG (blue), Airflow (brown), thorax movement (orange), oxygen saturation (purple) and QI (red). (**A**,**B**) Plot of 23 consecutive heartbeats. The upper-most signal is the start, the bottom-most is the end of the sequence. The signals are synchronized and the maximum of R peak in ECG signal is situated at 0 ms. A: In the ECG signal, atrial fibrillation is shown. (**B**) Slightly differing height and chronological appearance of ventricular mechanical contraction in amplitude sequence can be seen. (**C**) The recorded signal during polysomnography is plotted. Flow and thoracic movement show normal movement and oxygen saturation is always in the upper 90%. A continuous measurement in BCG is possible, but the signal in the ECG indicates an absolute arrhythmia due to atrial fibrillation. (**D**) The diagram shows heartbeat cycle length of ECG and corresponding estimated BCG cycle length, as well as the calculated QI are shown. For the most time –during AF – ECG and estimated cycle length of the BCG signal correlate well with a near perfect overlap of the ECG (green) and BCG (blue) line. For heartbeat 3 and 17 the estimated cycle length by the BCG algorithm differs strongly from the ECG. For these cases, the QI decreases, indicated by a dip of the red line at these heartbeats. However, due to the flexible algorithm the following heartbeats are calculated correctly.

Calculating descriptive statistical parameters estimated for each polysomnography recording per participant, we could demonstrate heterogeneity among the individual recordings (supplementary material Table S1, Fig. S1). Reasons for episodes of bad signal quality in participants were mainly caused by intervals of poor placement of the upper body on the sensor or increased body movement. In rare cases, bad signal quality was due to episodes of arrhythmia with frequent beat-to-beat changes of the BCG amplitude pattern like premature ventricular contractions or AV node conduction blocks (supplementary material Figs S2–S4). Premature supraventricular contractions or atrial fibrillation (Fig. 4) seemed to slightly decrease the signal quality and in rare cases accuracy of cycle length estimation but the algorithm could still provide good coverage and reliable measurements. Trustworthiness of the QI for estimation of the cycle length is demonstrated in supplementary material Fig. S5 by a high correlation the calculated correlation coefficient between ECG and BCG per participant.

**Figure 5:**
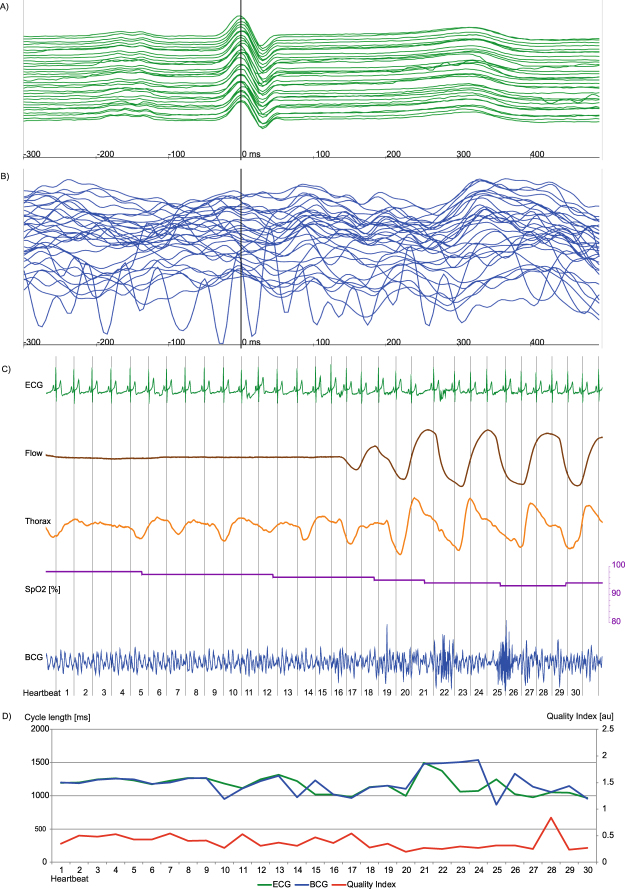
Episode of obstructive sleeping apnea with acceptable signal correlation. While the estimator calculates a good BCG to ECG correlation during the apnea phase, the phase of arousal is associated with increased muscular tone leading to a BCG to ECG mismatch and a decreased QI (Fig. 5D). Each signal is visualized by a unique colour: ECG (green), BCG (blue), Airflow (brown), thorax movement (orange), oxygen saturation (purple) and QI (red). (**A**,**B**) Plot of 32 consecutive heartbeats. The upper-most signal is the start, the bottom-most is the end of the sequence. The signals are synchronized and the maximum of R peak in ECG signal is situated at 0 ms. While the ECG is virtually not affected by the increased muscular tone of the phase of arousal, the BCG analysis is hampered by a strong superposition of mechanic heart activity. A: P-waves can be identified. In the lower third of the plot the ECG signal is slightly affected by increased body movement. B: The BCG signal suffers from strong deterioration due to body movement during this episode of obstructive sleeping apnea. At the upper half, a specific BCG sequence can be identified whereas at the lower half the identification of a decent amplitude sequence becomes more difficult. (**C**) Plot with simultaneously recorded ECG, air flow (brown), thoracic movement (orange, oxygen saturation (purple) and BCG (blue) deflection. There is nearly no flow due to the obstruction of the upper airway indicated by a near flatline in the flow signal. With decreasing oxygen saturation, thoracic movement increases and leads to an arousal-correlated reaction with increased muscular tone and superposition of mechanic heart activity. (**D**) A continuous BCG measurement is feasible but signal analysis is hampered during arousal and lacks good signal correlation in heartbeats 10, 14, 15, 20–29, indicated by a low QI. As soon as the muscular tone decreases, the algorithm again offers good BCG signal analysis indicated by a good QI in heartbeat 30.

**Figure 6:**
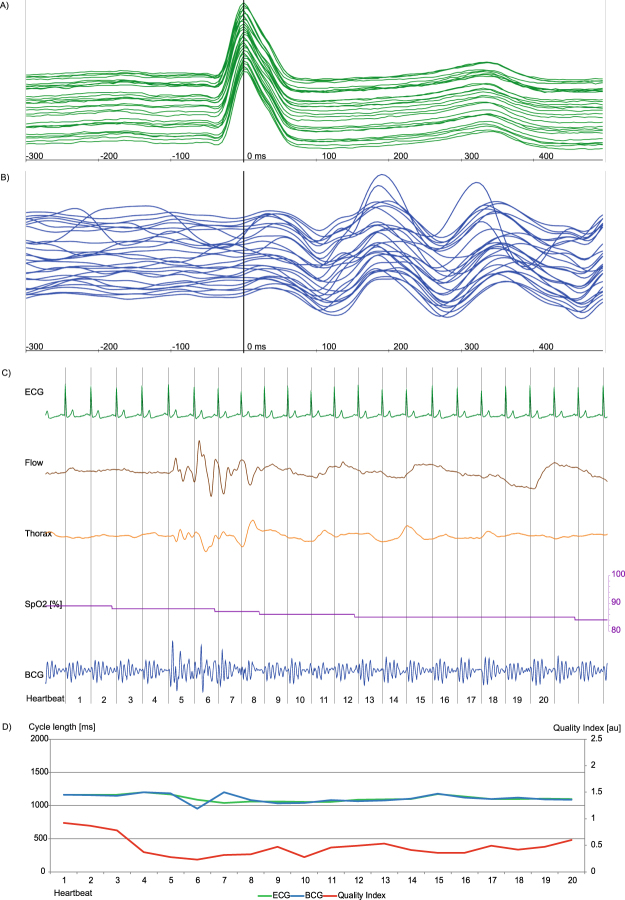
Episode of central sleeping apnea with good signal correlation. Arousal and the following deep respiration manoeuvres are associated with increased muscular tone and subsequently lead to decreased BCG signal detection in comparison to the simultaneously recorded ECG with slight cycle length mismatch and decreased QI. Each signal is visualized by a unique colour: ECG (green), BCG (blue), Airflow (brown), thorax movement (orange), oxygen saturation (purple) and QI (red). (**A**,**B**) Plot of 23 consecutive heartbeats. The upper-most signal is the start, the bottom-most is the end of the sequence. The signals are synchronized and the maximum of R peak in ECG signal is situated at 0 ms. A: In the ECG signal a sinus rhythm can be identified. (**B**) The BCG shows acceptable overlap in amplitude height and chronological sequence. In contrast to the BCG during obstructive sleeping apnea (Fig. 5) the signal is more harmonic with a slight superposition during deeper respiration manoeuvres. (**C**) Plot with simultaneously recorded ECG (green), air flow (brown), thoracic (orange) movement amplitude, oxygen saturation (purple) and BCG (blue) deflection. In the apnea phase, there is nearly no flow and no thoracic- or abdominal-movement. With decreasing oxygen saturation, thoracic- and abdominal-movement are increasing leading to an arousal correlated reaction with increased muscular tone and superposition of the mechanic heart activity in the recorded BCG signal. A continuous BCG measurement is feasible. But signal analysis is hampered in beats 5–8 showing moderate signal correlation indicated by a lower QI.

Examples of BCG signal analyses are displayed in Figs 4,5 and 6 and in supplementary material Figs S2,S3 and S4. Figure 4 shows an episode of AF with overall good signal analysis and good ECG and BCG cycle length correlation, interrupted by very long cycle lengths with altered electro-mechanical contraction and resulting poor accurateness of BCG cycle length estimation. In Fig. 5, an obstructive apnea episode is shown by decreased oxygen saturation associated with an arousal reaction and increased muscular tone. The superposition of muscular tone and body movement hampered signal analysis, as reflected by a decreased QI, which allowed filtering out these episodes. Once these episodes passed, signal analysis with a good QI resumed through the flexible peak pattern analysis of the algorithm. In Fig. 6 an episode of central apnea is shown with moderate to good signal coverage. In supplementary material Fig. S2 an episode of intermittent AV node block is shown with good coverage and accurateness, but problems in BCG cycle length estimation during the beats with blocked conduction in AV node. Whereas in supplementary material Fig. S3 the same patient at a later time point suffered from a persistent 2:1 AV node conduction block with better BCG cycle length estimation because the heartbeats do not differ as much in beat-to-beat comparison. In supplementary material Fig. S4 a difficult measurement situation is presented in a participant with BMI 45.9 kg/m^2^ suffering from sinus arrhythmia and a premature ventricular contraction during an episode of severe obstructive sleep apnea. Two heartbeats were excluded automatically by the algorithm, because the QI was to poor for reliable cycle length estimation.

## Discussion

The present study demonstrates the feasibility of long-term heartbeat interval detection by a ballistocardiographic sensor foil and a novel beat-to-beat algorithm during in-laboratory polysomnographic diagnostic.

Participants in our study were suspected to suffer from sleep disordered breathing, an illness which may be accompanied by snoring and strong movements during arousal events. Thus, artefacts caused by non-heartbeat movements were a concern for signal recording. In addition, the mean BMI of the patient cohort was 33.8 ± 9.6 kg/m^2^ with a maximum weight of 225 kg, potentially further lowering signal amplitude. Episodes with no signal or low signal quality, as well as all movements other than those caused by mechanic heart activity like respiration, were classified as artefacts. After automatic exclusion of these artefacts, the algorithm provided a moderate to good baseline correlation coefficient of *r*_*s*_ = 0.77. By further filtering the signal with the help of an automatically computed QI for each identified heartbeat, we were able to raise BCG cycle length estimation to a good to very good correlation of *r*_*s*_ = 0.95, however lowering the number of analysable heartbeats.

There were several novel aspects in this approach in addition to our previous work. To simulate a low or non-medically controlled environment, BCG recording was surveyed in a control room, but it was not allowed to enter the polysomnography room and to improve the recording after initial placement of the patient on the sensor and initiation of the measurement. BCG recording continued in episodes of reduced signal quality or while the patient left the bed. Our algorithm identified these episodes of no signal or low signal quality and excluded them automatically from baseline analysis. The accuracy of the calculated QI to identify heartbeats with good cycle length estimation is shown by Fig. 3 and the strong relationship of the QI with the calculated correlation coefficient between ECG cycle length and estimated BCG cycle length (supplementary material Fig. S5) could be demonstrated. The system provided robust and reliable measurements in different heart rhythms, despite superposition of the BCG signal due to sleep disordered breathing for hours of continuous measurement. Additionally, thanks to advances in our algorithm in combination with the BCG sensor, the field of potential applications was broadened for more different environments, diseases and lengths of recording.

Our system appears to work best for heartbeat cycle lengths within physiological parameters (Table 3) irrespective of the underlying heart rhythm (Figs 4,5 and 6, supplementary material Fig. S3). However, it could provide near perfect cycle length estimation, compared to a synchronized ECG. BCG recording appears to be suitable for a medically low- or uncontrolled environment or in combination with other measurements as an opportunity to record heart rate. The BCG algorithm offered robust and reliable results for different heart rhythms, which potentially offers future application where unobtrusive and long-term recordings can improve diagnosis and therapy. Indeed, this set up may be worthwhile – beside typical cardiac applications – for all kinds of diseases, which are associated with arrhythmia or where heart rate control is an aspect of treatment. Although ECG is the current “gold standard” for heartbeat evaluation, there are limits to the technology as well as its usability. Furthermore, it is not best suited for measurement situation, where unobtrusive monitoring is required. For BCG measurements, the patient’s chest merely needs to be placed above the BCG foil, a feasible technique in a low- or uncontrolled medical environment.

On the other hand, limits of the algorithm became clearer in this long-term recording as the algorithm estimated cycle length for heartbeats with a distinctive change of amplitude pattern in rare cases of poor correlation with real heartbeat cycle length in ECG (supplementary material Figs S2 and S4). The QI allowed to exclude these heartbeats easily, but for patients suffering from frequent beat-to-beat changes the algorithm at the current stage of development may not provide throughout an acceptable measurement. The estimated extreme cycle lengths (Table 3, Figs 2 and 3) were in some cases caused by these incorrect calculations. To clarify the accuracy of the algorithm in very fast and very slow heart rates further testing will have to be conducted. Altogether, misinterpretation of heartbeats was limited to some premature ventricular contractions and to episodes of intermittent AV node conduction blocks (supplementary material Figs S2 and S4). Atrial fibrillation (Fig. 4), sinus brady-, tachy- and arrhythmia, atrial tachycardia as well as premature supraventricular contraction did not seem to hamper the working of the algorithm considerable. In this feasibility study, the number of patients included was low, but the number of analysed heartbeats was high. Due to the low number of participants, QI values must be validated in a cohort with more participants. To analyse organ activity, the algorithm must know the frequency range of interest. We used 30 to 180 heartbeats per minute. Hence, 53 heartbeats faster and 57 heartbeats slower than these limits could not be interpreted correctly and were thus excluded. This corresponds to roughly only <0.0003% of all analysed heartbeats.

For future applications, a signal related to respiration – considered an artefact in the current setting – would be a desirable additional parameter, which could be potentially extracted from the BCG signal. From a technical point of view, we envision the presented algorithm as the first stage of a more comprehensive method that takes not only cycle length but also the amplitude and changes in the waveform pattern into account. In the past, we had positive results with using machine learning algorithms trained to distinguish AF and sinus rhythm based on amplitude and periodicity features extracted from the BCG. This earlier work was limited in that we did not yet have the beat-to-beat cycles available, so a natural next step would be to investigate a combination of these methods^24^. Another direction for future technical work would be the use of multiple spatially distributed BCG sensors. Here also, we could report some initial success on sinus rhythm patients that make us optimistic that there is good potential for reliable rhythm discrimination based on such a system^25^.

We conclude that a long-term beat-to-beat cycle length analysis with our novel algorithm using a BCG sensor is feasible, providing good heartbeat detection and correlation with a simultaneously recorded ECG even in a setting with multiple artefacts and signal superposition.

## Methods

This investigation was performed at the Department of Neurology, University Hospital RWTH Aachen, Germany. Ethical and regulatory classification according to German medical device act and “Berufsordnung Ärzte (BOÄ)” was performed by the coordination center for cardiology studies at the University Hospital of Aachen. The study was approved by the institutional review board of the University Hospital Aachen (Registration number: EK204/11, date: 27.05.2011; Clinical trials gov.: NCT 01775241). The study met the ethical principles of the Declaration of Helsinki, the guidelines of Good Clinical Practice and the current legal requirements.

### Study design and Study population

We included 21 patients after positive screening for sleep disordered breathing. The mean AHI was 40.9 ± 9.6 /h and ESS 13 ± 5. All participants were scheduled for overnight diagnostic PSG. The BCG and PSG measurements were performed in the Sleep Center at the Department of Neurology, University Hospital RWTH Aachen, Germany. Written informed consent was obtained for all participants prior to commencement of the study according to the following criteria: Patients suspected for sleep breathing disorder and scheduled diagnostic PSG, at least 18 years of age and ability to understand the investigation and to follow the instructions of study staff. Exclusion criteria were: Pregnancy or lactating and mentally incapacitated persons.

### Data acquisition and analysis

Overnight diagnostic PSG was performed with a digital polygraph of Schwarzer Brainlab (Schwarzer Brainlab, Software Version 4.00, Munich, Germany; Barcelona: Deltamed, Software version 2007 Paris, France). Noninvasive HR measurement was performed during polysomnography with a BCG foil (30 × 60 cm) of EmFit (EmFit Ltd. Vaajakoski, Finland). Electrical safety was approved by the VDE (“VDE Verband der Elektrotechnik, Elektronik, Informationstechnik e.V.”, Frankfurt, Germany) according to EN IEC 60601-1.

### Ballistocardiographic sensor

The EmFit sensor is a thin and flexible foil consisting of charged polymer layers containing air voids that react like an electrical capacitator. Mechanical pressure on the foil changes the position and geometry of the air voids with respect to each other resulting in a shift of the electrical charge. This shift of the electrical charge can be measured and converted to a voltage signal. Any mechanical activity on the sensor by inner organs and muscular movement deforming the skin^26^ is part of the recorded signal. To improve signal quality, the sensor should be placed next to the organ of interest and if possible perpendicular to its main movement axis. For a ballistocardiographic measurement, we placed the BCG foil under the chest of the participant during PSG in supine position without direct contact with the sensor (Fig. 1A). Thus, the measurement is performed in a dorsoventricular axis. This may not be considered as the ideal direction to record the highest amplitude in BCG deflection but the most convenient for long term unobtrusive recording. Deformation of the BCG foil by the mechanical heart contraction moves the charged air voids in the BCG foil in respect to each other (Fig. 1B,C) generating an electrical charge which can be recorded and displayed in an ECG related signal (Fig. 1D). The signal was acquired with 1000 Hz and our algorithm offers near real-time analysis with a latency of less than 2 seconds.

### Signal processing

**Figure 7:**
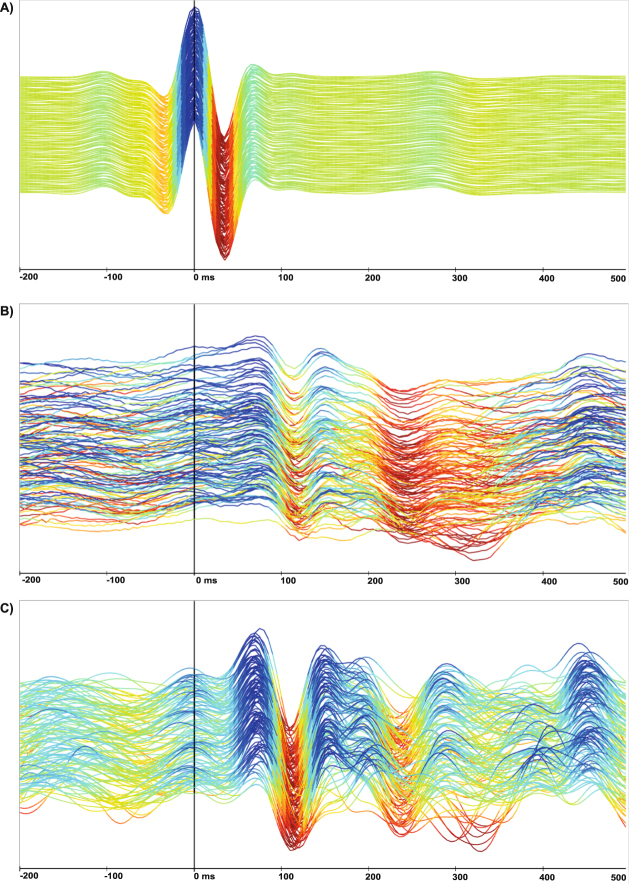
Diagram of 128 consecutive heartbeats in ECG (**A**) and BCG (**B**,**C**) recording with time dependent analysis by ECG R-wave peak detection at 0 ms in any diagram. For better amplitude visualization, the maxima and minima are visualized with colour gradients in an arbitrary scale; (**A**) The ECG shows a harmonic signal over the whole displayed time during sinus rhythm; (**B**) Sinus rhythm; genuine BCG signal deteriorates due to signal superposition of breathing and movement artefacts. (**C**) Sinus rhythm (filtering the BCG signal) exposes a typical mechanical amplitudes sequence of each heartbeat but also shows the ambiguous and intraindividually variable nature of mechanical heartbeat movement.

ECG signals are distinguished by a heart rate-dependent sequence of a distinct electrical deflection. In Fig. 7A, an example ECG episode of randomly chosen 128 consecutive heartbeats is shown, the R peak is marked at 0 ms at the time-scale. All consecutive heartbeats are near identical. Due to the superposition of the BCG signal with any kind of mechanical pressure on the EmFit foil, like cardiac, breathing or other mechanical activities (Fig. 7B), the genuine recorded signal shows far wider variability in time and shape and has to be further processed to extract the cardiac movement as surrogate for mechanical heart activity. After filtering motion artefacts and superimposing breathing activity, a regularly repeating sequence of peaks becomes visible (Fig. 7C). In contrast to an ECG, there is again high inter- and intrapatient variability in BCG signal pattern, which makes an automated signal detection more complicated (supplementary material Fig. S6A–D).

**Figure 8:**
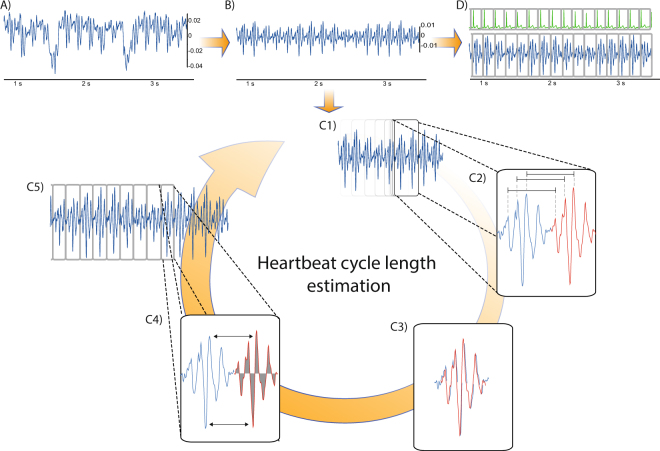
Processing the genuine BCG signal, identifying and analysing the heartbeats and correlation with a simultaneous recorded ECG. (**A**) Genuine BCG signal shows a superposition of slow oscillating respiration work at 5–10 seconds, high but smaller oscillating cardiac activity as well as deterioration by artefacts like body movement (**B**) After filtering the respiration and movement artefacts, the mechanical vibration of cardiac activity becomes visible; (C1) A sliding window of interest moves forward on the signal and identifies artefacts and marks corrupted sequences or continues for further analysis. (C2) By comparison of repeating amplitude sequences, consecutive heartbeats (blue and red line) are identified in a frequency range of interest; (C3) Three local interval estimators compare the two identified BCG patterns for their specific sequence, height, area under the curve and cycle length. (C4) Out of this information an individual cycle length and QI of each captured beat is estimated. The QI shows the comparability to the previous identified beats and allows to filter artefacts; (C5) Estimated parameters of each identified heartbeat are stored for further analysis. (**D**) Heartbeat cycle length is correlated for this study with a simultaneously recorded ECG.

To prepare the BCG signal for further analysis, specific steps of preprocessing have to be performed. The genuine recorded EmFit signal (Fig. 8A), recorded in the dorsoventricular axis is superimposed by breathing and other organ activity or body movement. In the vertical axis, large oscillations with a wavelength of 5 to 10 seconds are visible corresponding to respiratory work. Within these respiration waves, smaller oscillations with a higher frequency are visible but seem to show no specific sequence. After applying a time-domain filter and differencing the signal, breathing artefacts are removed and a repeated specific sequence related to the cardiac expulsion of blood in the big arteries^27^ as surrogate for the cardiac mechanical activity is uncovered (Fig. 8B). We used identical filter settings for all recordings with 80 dB stop-band attenuation and a cut-off frequency of 0.5 Hz.

### Beat-to-Beat analysis

After pre-processing, each heartbeat must be identified and analysed by a forward moving window of interest (Fig. 8C1–C5). The extracted cardiac mechanical activity, when compared to a synchronized recorded ECG, shows a wider variability in time, amplitude height, chronology of peaks and overall shape. Commonly used techniques for automated cardiac signal detection work with a fiducial point analysis to identify previous well-known deflections or peak patterns, such as the QRS complex in the ECG. Due to the ambiguous character of the BCG deflection with high inter- and intrapersonal variability (supplementary material Figs S1 and S6A–D), a fiducial point analysis does not seem feasible for BCG analysis^12^. Thus, a novel algorithm inspired by the pitch-tracking method of speech analysis was used^23,28^ for a beat-to-beat analysis and to determine the cycle length, specific signal quality and other parameters of each identified heartbeat. With a window of interest, the signal is scanned for predefined frequencies. By this approach, the specific peak pattern does not have to be known beforehand, but we assume that consecutive heartbeats consist of corresponding sequences of amplitudes.

The window of interest is more than twice the length of the lowest expected cycle length and uses a stepwise approach of 200 ms forward in time. For each new position, an adaptive threshold measurement of amplitude is performed (Fig. 8C1). In case of violation of the threshold, this timeframe is considered as corrupted by a high-energy artefact and ignored in further analysis. The window of interest then moves on and starts a new measurement. If there is no threshold violation in the analysed window of interest, the algorithm continues with the comparison of repeating amplitude patterns (Fig. 8C2, indicated by a blue and red line).

Three local interval estimators compare the actual sequence (red line) to the preceding heartbeat (blue line) calculating the heartbeat specific cycle length, integral of curve and amplitude height (Fig. 8C3) and a resulting QI (Fig. 8C4). To validate the calculated cycle length in the BCG we applied the “Open Source Arrhythmia Detection Software” (EP Limited, 35 Medford St., Somerville, MA, USA) to a synchronized recorded ECG (Fig. 8D). For validation, we considered only episodes of at least 10 consecutive heartbeats with a least a QI of 0.1.

### Calculating the Quality Index (QI)

Due to the fact that the genuine BCG signal itself is sometimes difficult to interpret if the recorded signal quality is poor or superimposed by artefacts, we added a QI. The QI allows the algorithm and users to classify the identified heartbeat in BCG signal and the estimated cycle length for its reliability and potential accurateness. Thus, the QI marks episodes of low or no signal quality to identify episodes with artefacts and to automatically exclude them from analysis due to the assumption of low accurateness of cycle length estimation for these episodes.

On a conceptual level, the QI is a measure of how similar the waveforms of two detected subsequent heartbeats are to each other. The underlying intuition behind the method is that if two subsequent heartbeats identified by the algorithm are highly dissimilar, there also is a high chance that at least one of these beats is in fact an artefact and the resulting cycle length invalid.

Technically, the QI describes the likelihood of the estimated cycle length after a Bayesian fusion of the three different local interval estimators on an arbitrary scale. If the three estimators disagree with each other on the most likely cycle length, the resulting QI would attain a value close to zero, whereas a higher accordance among the local interval estimators leads to a higher QI, which should result in a more precise calculation of this single heartbeat’s characteristics^23,28^. Additionally, identifying artefacts by the adaptive threshold comparing the QI allows to filter episodes of low signal quality as well as to improve the accuracy of cycle length detection at the cost of a reduced absolute number of analysable heartbeat episodes. The calculations of each identified heartbeat are stored for further correlation analysis (Fig. 8C5).

### Statistical analysis

Distribution of the recorded ECG and BCG heartbeats showed right-sided outliers, leading to skewness to the left and increased kurtosis. In time-series analysis, considerable autocorrelation was present in the data. For such a big sample of auto-correlated measurements with inter-participant heterogeneity, conventional significance tests for normality are not valid. Therefore, we analysed the data predominantly with descriptive methods like histograms, Bland-Altman and scatter plots. Because of the outliers, Spearman rank correlation was preferred to the Pearson coefficient. To avoid outlier effects, in some cases we used the median to represent the central tendency of the recorded data. For qualitative analysis, all values are expressed in percentages and absolute numbers. Statistical analysis was performed with SPSS 24 (IBM Corp. Released 2016. IBM SPSS Statistics for Windows, Version 21.0. Armonk, NY: IBM Corp.). ECG and BCG deflections were analysed and displayed with Signalplant 1.2.2.4 (MEDISIG – F. Plesinger; J. Jurco; 2013–2017; Institute of Scientific Instruments of the CAS; Czech Republic). Although our algorithm needs no training phase, the QI was calculated by comparing at least two consecutive heartbeats. Thus, we applied the algorithm to the complete BCG signal, but considered for statistical analysis only episodes of at least 10 consecutive heartbeats with a QI > 0.1.

### Data availability statement

The datasets generated during and/or analysed during the current study are available from the corresponding author upon reasonable request.

## Electronic supplementary material

Supplementary Material
