# Supplementary material for: Unobtrusive Nocturnal Heartbeat Monitoring by a Ballistocardiographic Sensor in Patients with Sleep Disordered Breathing
Source: Sci Rep. 2017 Oct 13;7:13175. doi: 10.1038/s41598-017-13138-0 (PMC5640641; doi:10.1038/s41598-017-13138-0)
Supplement: Supplementary file 1 — Supplementary Material [file 41598_2017_13138_MOESM1_ESM.doc]

*Supplementary Material*

**Unobtrusive Nocturnal Heartbeat Monitoring by a Ballistocardiographic Sensor in Patients with Sleep Disordered Breathing**

**Unobtrusive Nocturnal heartbeat monitoring**

*Matthias Daniel Zink*¹, MD; Christoph Brüser^2^, PhD; Björn-Ole Stüben¹; Andreas Napp^1^, MD; Robert Stöhr¹, MD, PhD; Steffen Leonhardt^2^, MD, PhD; Nikolaus Marx¹, MD; Karl Mischke¹, MD; Jörg B. Schulz^3, 4^, MD; Johannes Schiefer^3^, MD*

*¹Department of Cardiology, Pneumology, Angiology and Intensive Care Medicine, University Hospital RWTH Aachen, Pauwelsstr. 30; 52074 Aachen; Germany*

*^2^Philips Chair for Medical Information Technology, Helmholtz-Institute, RWTH Aachen, Pauwelsstr. 20, 52074 Aachen, Germany*

*^3^Department of Neurology, University Hospital RWTH Aachen, Pauwelsstr. 30; 52074 Aachen; Germany*

*^4^Jülich Aachen Research Alliance (JARA) – JARA-Institute Molecular Neuroscience and Neuroimaging, FZ Jülich and RWTH University*

|  | Mean | | | | Median | | | |  |
| --- | --- | --- | --- | --- | --- | --- | --- | --- | --- |
| Patient | Quality Index [au] | ECG [ms] | BCG [ms] | ECG-BCG [ms] | Quality Index [au] | ECG [ms] | BCG [ms] | ECG-BCG [ms] | *r_s_* |
| 1 | 0.59 | 893 | 876 | 16.82 | 0.57 | 860 | 860 | 1 | 0.84 |
| 2 | 0.45 | 898 | 836 | 61.8 | 0.36 | 870 | 846 | 4 | 0.38 |
| 3 | 0.42 | 1037 | 1035 | 2.67 | 0.33 | 1020 | 1026 | 0 | 0.42 |
| 4 | 0.39 | 764 | 773 | -9.23 | 0.31 | 730 | 747 | 0 | 0.27 |
| 5 | 0.62 | 1723 | 1333 | 389.39 | 0.43 | 1755 | 1217 | 10 | -0.12 |
| 6 | 0.55 | 947 | 940 | 6.19 | 0.52 | 940 | 941 | 3 | 0.88 |
| 7 | 0.52 | 745 | 784 | -38.65 | 0.38 | 680 | 765 | -1 | 0.48 |
| 8 | 0.87 | 881 | 879 | 1.73 | 0.84 | 875 | 867 | 0 | 0.93 |
| 9 | 0.6 | 1012 | 996 | 15.22 | 0.47 | 1010 | 997 | 1 | 0.62 |
| 10 | 0.64 | 1130 | 1128 | 1.57 | 0.55 | 1130 | 1130 | 1 | 0.8 |
| 11 | 0.84 | 835 | 832 | 3.41 | 0.81 | 835 | 836 | 0 | 0.94 |
| 12 | 0.37 | 766 | 796 | -30.06 | 0.33 | 770 | 787 | -7 | 0.17 |
| 13 | 0.5 | 1012 | 1014 | -1.9 | 0.39 | 1000 | 1006 | 0 | 0.65 |
| 14 | 0.57 | 996 | 993 | 2.61 | 0.48 | 995 | 995 | 1 | 0.71 |
| 15 | 0.33 | 1174 | 1165 | 8.29 | 0.29 | 1150 | 1162 | 4 | 0.51 |
| 16 | 0.81 | 899 | 889 | 10.53 | 0.73 | 895 | 895 | 1 | 0.92 |
| 17 | 0.66 | 693 | 722 | -29.65 | 0.49 | 680 | 696 | 0 | 0.47 |
| 18 | 0.55 | 1101 | 1094 | 7.71 | 0.49 | 1090 | 1080 | 1 | 0.83 |
| 19 | 0.62 | 848 | 832 | 16.12 | 0.5 | 840 | 834 | 2 | 0.67 |
| 20 | 0.55 | 725 | 749 | -24.51 | 0.47 | 720 | 738 | 0 | 0.52 |
| 21 | 0.66 | 919 | 911 | 8.00 | 0.63 | 920 | 919 | 0 | 0.79 |

**Table S 1 Shows the arithmetic mean and median for the Quality Index, the calculated ECG and the estimated BCG cycle length as well as the differences of ECG and BCG cycle length per participant.** The rightmost column gives the Spearman rank correlation (r_s_) between the ECG and BCG time series per participant. Mean ECG-BCG differences are strongly influenced by outliers of several participants, whereas the median is very close to zero in almost all participants – in combination with the substantial Spearman rank correlations – indicating close agreement between both measurements.


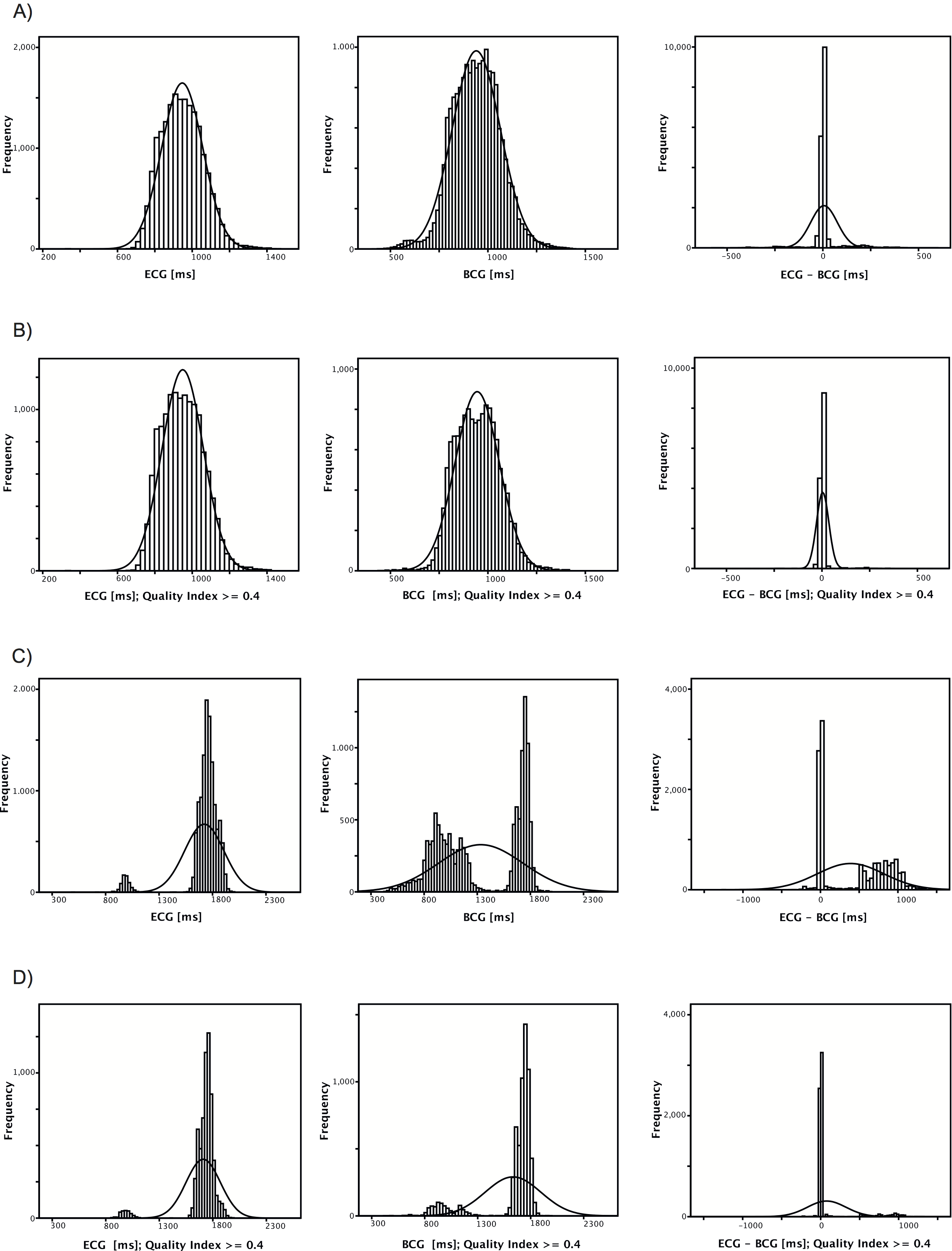


**Figure S 1 Histograms from left to right of the ECG, estimated BCG cycle lengths and the difference between ECG and BCG cycle length per time point. A:** Shows the measurements of a patient with a good baseline correlation. The histograms are normally distributed and look similar in shape. In the histogram of the difference there is an accumulation around zero difference between the ECG and estimated BCG cycle lengths. **B:** Shows the same data but filtered for heartbeats with a quality index ≥ 0.4. There is even more overlap in shape between the ECG and estimated BCG cycle length as well as a sharper concentration of the difference around zero and a reduction of outliers. **C:** Shows data of the patient with the worst measurement. The measurement was hampered due to intermittent premature ventricular contractions, episodes of AV node conduction block as well as intermittent very low signal quality due to bad contact to the sensor and artefacts by body movement. The ECG histogram indicates a two-headed distribution as a result of arrhythmia. The baseline BCG histogram shows the same two headed distribution, but with incorrect estimated heartbeats between 800 and 1300 ms. This incorrect cycle length calculation can be seen as well in the histogram of the differences on the right side as the accumulation between +500 and +1000 ms. **D:** By filtering this data for heartbeats with a quality index ≥ 0.4, ECG and estimated BCG cycle lengths show a better overlap in shape. Events of arrhythmia are still present visualized by the accumulation of heartbeats with a cycle length between 800 and 1300 ms. In the histogram of the differences, the accumulation around zero is near equal to the unfiltered histogram but the incorrect calculated heartbeats between +500 and +1000 ms are almost excluded.

**Figure S 2** **Shows an episode of intermittent AV node conduction block.** Each signal is visualized by a unique colour: ECG (green), BCG (blue), Airflow (brown), thorax movement (orange), oxygen saturation (purple) and quality index (red).

**A and B:** Plot of 22 consecutive heartbeats. The upper-most signal is the start, the bottom-most is the end of the sequence. The signals are synchronized and the maximum of R peak in ECG signal is situated at 0 ms. **A:** In the ECG signal, a sinus rhythm is shown, with an intermittent AV node conduction block. B: The BCG shows an acceptable overlap in amplitude height and chronological sequence interrupted by some slight time-shifts.

**C and D:** Plot with simultaneously recorded ECG (green), air flow (brown), thoracic (orange) movement amplitude, oxygen saturation (purple) and BCG (blue) deflection. In case of AV node conduction block (heartbeat 3, 10 and 18) the algorithm struggled estimating a correct heartbeat cycle length with a subsequent decrease in quality index. For sinus rhythm heartbeats in between the algorithm provides a near perfect correlation to the ECG and calculates for these heartbeats also high to very high quality indices (heartbeats 6-8 and 12-14).

**Figure S 3 Shows the same participant at a later time point with persistent AV node conduction block.** Each signal is visualized by a unique colour: ECG (green), BCG (blue), Airflow (brown), thorax movement (orange), oxygen saturation (purple) and quality index (red).

**A and B:** Plot of 21 consecutive heartbeats. The upper-most signal is the start, the bottom-most is the end of the sequence. The signals are synchronized and the maximum of R peak in ECG signal is situated at 0 ms. **A:** In ECG signal, a persistent AV node 2:1 conduction block is shown. **B:** BCG signal shows a good overlap in amplitude pattern.

**C and D:** Plot with simultaneously recorded ECG (green), air flow (brown), thoracic (orange) movement amplitude, oxygen saturation (purple) and BCG (blue) deflection. Due to the almost constant shape in BCG amplitude pattern the algorithm provided a near perfect correlation (overlap of blue and green line, Fig. S 3 D) with high quality index (red line, Fig. S 3 D).

**Figure S 4** **Shows a difficult measurement scenario in a patient with a BMI of 45.9 kg/m^2^ suffering of an episode of obstructive sleep apnea with high body and thoracic movement, additionally during sinus arrhythmia a premature ventricular contraction (heartbeat 24, Fig. S 4C&D) occurs.** Each signal is visualized by a unique colour: ECG (green), BCG (blue), Airflow (brown), thorax movement (orange), oxygen saturation (purple) and quality index (red).

**A and B:** Plot of 33 consecutive heartbeats. The upper-most signal is the start, the bottom-most is the end of the sequence. The signals are synchronized and the maximum of R peak in ECG signal is situated at 0 ms. **A:** In ECG signal, a sinus arrhythmia is shown with a premature ventricular contraction. **B:** BCG signal shows high variability in amplitude height and chronological sequence due to increased thoracic and body movement in an episode of obstructive sleep apnea.

**C and D:** Plot with simultaneously recorded ECG (green), air flow (brown), thoracic (orange) movement amplitude, oxygen saturation (purple) and BCG (blue) deflection. The algorithm excluded automatically two heartbeats (4 and 11) due to bad signal quality. During increased thoracic movement, the accurateness of BCG heartbeat cycle length estimation is bad visualized by the lack of overlap of the ECG and BCG line (green and blue line, Fig. S 4D) and low quality index (red line, Fig. S 4D).

**Figure S 5 Median of the quality index of each participant plotted against the correlation coefficient (r_s_) of each participant.** Showing a positive linear relationship of calculated quality index with the correlation of ECG and BCG cycle length. Elaborating the accuracy of indicating high correlation between ECG and BCG cycle length calculation by the quality index.

**Figure S 6** **Filtered BCG recordings (randomly selected 128 consecutive beats) at 0 ms on the timeline the corresponding R peak of the simultaneous recorded ECG is marked.** For better amplitude identification, the maxima and minima are coloured by an arbitrary scale. The BCG recordings show an increased inter- and intraindividual variability in amplitude pattern. **A-C:** Sinus rhythm, of three patients with their individual BCG signal pattern; **D:** BCG of a patient during an episode of atrial fibrillation
